# The realization of interpersonal meanings in cosmetic Maybelline New York in 2018 advertisements

**DOI:** 10.12688/f1000research.129750.1

**Published:** 2023-08-10

**Authors:** Herman Herman, Ridwin Purba, Nanda Saputra

**Affiliations:** 1 Department of English Education, Universitas HKBP Nommensen Pematangsiantar, Pematang Siantar, 21136, Indonesia; 2 Department of Teacher Profession Education, Universitas Simalungun, Pematang Siantar, 21139, Indonesia; 3 Department of Indonesian Education, STIT Al-Hilal Sigli, Aceh, 24114, Indonesia

**Keywords:** Interpersonal meaning, mood structure, advertisement, mood types, multimodal, systemic functional linguistics, metafunctions, Halliday

## Abstract

Background: Interpersonal meaning is the interaction between the speaker and the listener and the writer and the reader. This study aims to determine the structure of mood and how the structure of mood is found. The purposes of this study are (1) to be more sensitive to the mood structure shown in Maybelline New York cosmetic advertisement. (2) to investigate the reason of why interrogative sentences are more difficult to find than declarative sentences. Through this research, we can see the types of mood structures, and how these mood structures are used. Methods: The type of research approach used is descriptive qualitative. Research in this case describes systematically, factually and accurately facts and causal relationships of the phenomena studied. The sources of data in this study are eight cosmetic Maybelline New York products, namely the Maybelline gel pencil, L’oreal colour riche nail, Maybelline mascara and eyeliner, Maybelline creamy lipmatte and Maybelline eyeshadow, Maybelline eyeliner, Maybelline lipstick, and Maybelline clear smooth all in one. Results: In analyzing the data, the researcher of the 2014 Halliday theory revised by Mathiesse found that there are three types of manifested mood, four types of realized speech function, and mood-based realization of speech function. The moods manifested were declarative (48.64%), interrogative (5.40%) and imperative (45.94%). Speech functions that are realized are statements (48.64%), questions (5.40%), offers (18.91%) and command (24.32%). Conclusions: It can be concluded that declarative sentences are mostly found in Maybelline New York products. Researchers suggest that readers be more careful in using mood structures in cosmetic advertisements so that there are no misunderstandings.

## Introduction

Social beings utilize language as a tool to communicate with one another ( Lindsay and Knight, 2010; Herman *et al.*, 2020). For worldwide communication, English has grown in popularity and is frequently referred to as an international language. Language is necessary for people ( Eggins, 2004) to engage, communicate, and receive information from others.

Language is a means of communicating ( Butt, 2011; Herman, van Thao and Purba, 2021) with others through sending ideas, emotions, thoughts, information, and ideals ( Herman, van Thao and Purba, 2021). The process of conveying a person or a group's meanings, ideas, and understanding to another person or group is described as communication ( Sharma, 2017:259). The researcher draws the conclusion that communication is an interaction or relationship between 1-2 persons or a group of people in a society who share information. Text is used to communicate and the meaning and structure of the text are contained within it. Meaning (semantics) is crucial in this context because the goal of communication is for listeners to grasp what the speaker is saying.

The greatest level of language is semantics, which acts as a bridge between language and the outer world ( Halliday and Matthiesen, 2014:42). This indicates that semantics interfaces with content, but also with other systems that function within context. Lexicogrammar converts the linguistic meaning of experience and interpersonal relationships into words that reflect the speaker's perspective ( Halliday, 1994).

Therefore, researchers want to examine the interpersonal meaning in cosmetic advertising. Interpersonal meaning is one of the most fundamental interacting contrasts between using language to convey knowledge and using it to interchange products and services ( Susanto & Watik, 2017; Herman *et al.*, 2022c). Interaction between the speaker or writer and the listener or reader is thus tied to interpersonal meaning. Its purpose is to facilitate the interchange of rhetorical roles such as declarations, questions, offers, and commands ( Ngongo *et al.*, 2022).

Therefore, interpersonal meaning can also occur in an advertisement. Where interpersonal meaning is the interaction between the speaker and the listener and the writer and the reader ( Herman *et al.*, 2019). In this case, it can be seen that the relationship between interpersonal meanings and advertisements is very clearly related because in an advertisement there must be an author to write the advertisement and those who read the advertisement are the readers.

Advertisement is a process of conveying information about products such as goods, services, and ideas from a company to a target audience. Advertising is the origination or communication of product ideas to motivate consumers to purchase ( Belch & Michael, 2003). During the marketing process ( Goddard, 1998), advertisements can provide information, ideas or messages to the audience. The purpose of this process is to ask the audience to take action. Cosmetics sellers and commercial businesses require advertising services.

The purpose of cosmetic advertising is not only to provide information but also to introduce the product to buyers. Interpersonal meaning includes speech functions which include speech functions of statements, questions, offers, and commands and their realization in declarative, interrogative, and imperative atmospheres ( Herman *et al.*, 2022b).

Therefore, the researcher will examine a cosmetic advertisement product, namely the Maybelline gel pencil. The problem that occurs with the cosmetic advertisement of the product is that the ad does not explain in detail the target age for using these cosmetics. Which means, in the marketing of cosmetic advertisements, there must be a target age for who can use these cosmetic items. The second problem found was the lack of specificity in the delivery of uses and how to use these cosmetic items. What that means is that in an advertisement, whether it's a beauty advertisement, product advertisement, and so on, there must be a delivery of the use and instructions on how to use the beauty cosmetic item and when the beauty cosmetic item is used. The Maybelline gel pencil is useful for beautifying the eyebrows with color.

Most women use cosmetic products for their beauty so that their partner is comfortable with their beauty ( Mafra *et al.*, 2020) but do not see the side effects that will occur in the future. The side effects include facial wrinkles, acne, and blackheads. Examples of Maybelline New York advertisements are, Maybelline gel pencil, eyeliner, lipstick and powder.

Below is a picture of the Maybelline New York’s Gel Pencil beauty cosmetic advertisement:

**Figure 1.**
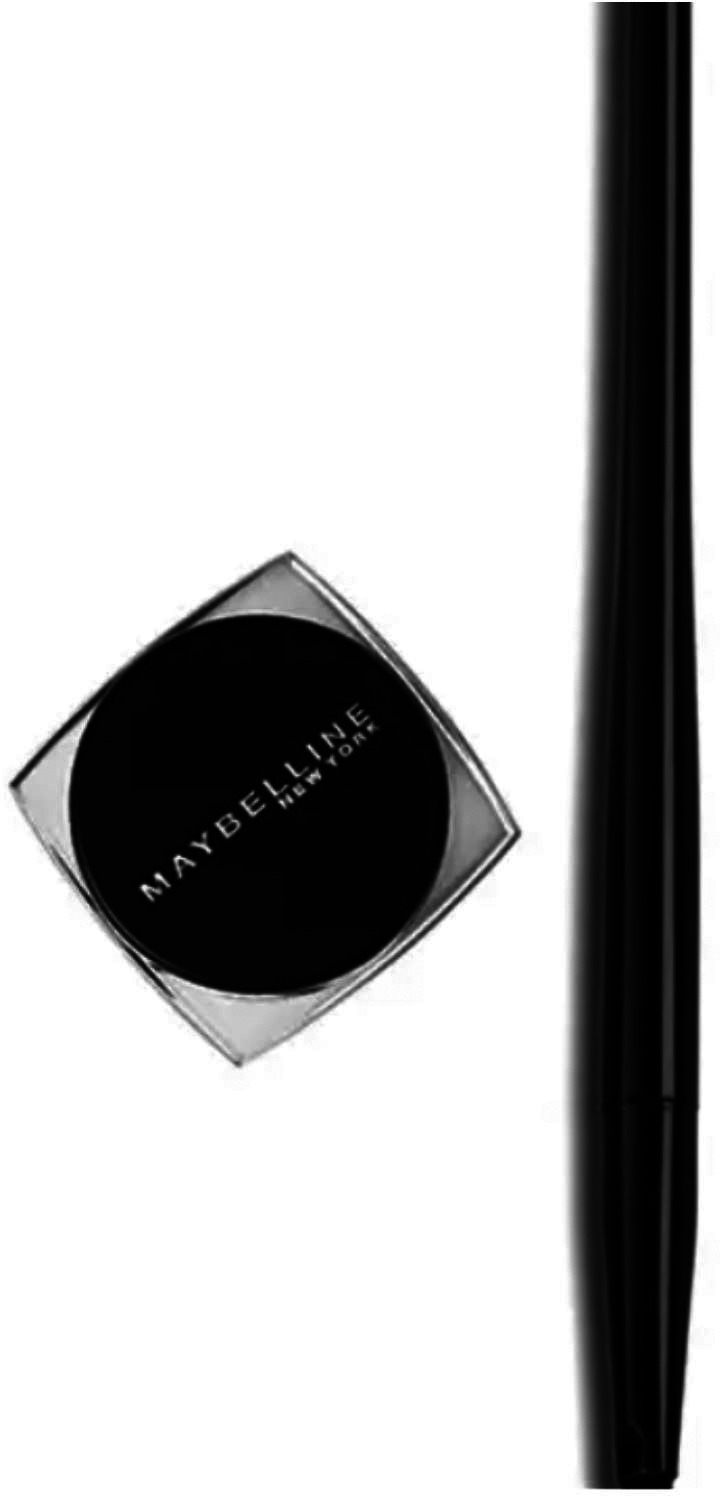
Maybelline New York’s Gel Pencil beauty cosmetic (Source: http://iprice.co.id/maybelline/makeup/mata/eyeliner/gel/).

From the example above, the researcher can see that the problem with the Maybelline gel pencil New York advertisement is that there is no delivery or instructions on how to use it. Based on the example above, we can clearly see the types of interpersonal meaning of the Maybelline gel pencil New York advertisement ( Tables 1- 4).

**Table 1.** You do get expert tips at maybelline.com ( https://www.maybelline.co.id/) [ Herman *et al.*, 2022a; Fang, 2016].

You	do	get	expert tips	at maybelline.com
Subject	finite	Predicator	Complement	Circumstantial: Place
Mood: Imperative		Residue		

**Table 2.** Now all-day intensity goes ultra smooth ( https://www.maybelline.co.id/) [ Herman *et al.*, 2022a; Fang, 2016].

Now all-day intensity	(does)	goes	ultra smooth
Subject	Finite	Predicator	Complement
Mood: Declarative		Residue	

**Table 3.** Maybe she’s born with it ( https://www.maybelline.co.id/) [ Herman *et al.*, 2022a; Fang, 2016].

Maybe	she	is	born	with it
Mood Adjunct	Subject	Finite	Predicator	Complement
	Mood: Declarative		Residue	

**Table 4.** Maybe it’s Maybelline ( https://www.maybelline.co.id/) ( Fang, 2016).

Maybe	it	is	maybelline
Mood Adjunct	Subject	Finite	Complement
	Mood: Declarative		Residue

In performing this research, the researcher draws on prior findings from Patpong's (2009) thesis ‘Thai Persuasive Discourse: A Systemic Functional Approach To An Analysis Of Amulet Advertisements’, which is related to this research. By examining three metafunctions, this study seeks to explain how persuasive discourse is lexicogrammatically investigated. According to the research findings, the relational process comes in second place to the material process in terms of ideational significance. Declarative mood is most frequently used in interpersonal contexts, followed by imperative. The unmarked topical theme was determined to be dominating (91.58%) in the textual analysis, followed by the labeled theme (8.42%).

As a result, it can be claimed that this research differs from earlier research. Cosmetic advertisements that contained four speech functions, including statements, questions, offers, and commands as well as three types of moods, including declarative, interrogative, and imperative in interpersonal meaning has been studied by Patpong. This study has only examined persuasive discourse in the previous study ( Patpong, 2009), which was investigated lexicogrammatically by investigating three metafunctions, namely ideational, interpersonal, and textual. As a result, the researcher concentrated only on 3 different moods and 4 speech functions in advertisement for beauty products.

The Halliday theory, proposed by Matthiessen in 2014, is in agreement with this research. Three different types of mood realization, four different types of speech function realization, and mood realization of speech function were discovered by the researcher. Declarative (83.33%), interrogative (3.33%), and imperative (13.33%) moods were realized. Statements (43.33%), questions (6.67%), offers (40%) and orders (10%) are the speech functions that are really used. This demonstrates that statements in advertising for goods are more likely to interest and persuade consumers.

The recent phenomenon in cosmetic advertising and the explanation given above is the reason why this research focuses on analyzing the type of interpersonal meaning in cosmetic advertisements.

## Methods

### Research design

This study was carried out utilizing a descriptive qualitative approach. Eight of the most popular methodologies, according to Ary *et al.* (2010:29), include the following: basic interpretative studies, case studies, document or content analysis, ethnography, grounded theory, historical studies, narrative inquiry, and phenomenological research. Because the researcher employed the scripts of advertisements as the subject of the investigation, this study utilized a document or content analysis. Content or document analysis, according to Ary *et al.* (2010:457), is a research technique used to analyze textual or visual materials with the goal of discovering certain qualities of the material ( Purba *et al.*, 2022). The source material for analysis could be a textbook. Because textbooks are collections of logical ideas that are organized according to learning objectives. This means that the objective of the product is given in the Maybelline New York cosmetics advertising, both on TV and in the catalog.

Sugiyono (2017) claims that qualitative researchers are human instruments that serve to state the research focus, choose information as a data source, compile data, assess data quality, analyze data, interpret data, and draw conclusions about findings ( Sugiyono, 2017 cited in Munthe *et al.*, 2021). The sources were gathered at random by searching for advertisements on the internet. The researcher utilized the internet, YouTube, a phone, a television, a pen, a book, and a piece of data in this study as the tool to get the data. The data were collected using observational methods. Google was used to search for and download the complete text of advertisements. Next, we downloaded cosmetics advertisement texts that only used English as the language of communication. A detailed explanation of the data collection and research instruments is provided in the data collection section. Watching product ads on YouTube can be a more effective way to gain insight into a product. We were able to quickly and easily filter search results to find the specific product they are interested in. YouTube offers extra information about the product being advertised. This includes links to websites where viewers can purchase the product and read more reviews.

### Data sources

Data are units of information, often numeric, that are collected through observation. Burnham (2012:1) stated that research data is the information that is typically kept and acknowledged by the scientific community as being required to validate research findings. The sources of data in this study are eight cosmetic Maybelline New York products, namely the Maybelline gel pencil, L’oreal colour riche nail, Maybelline mascara and eyeliner, Maybelline creamy lipmatte and Maybelline eyeshadow, Maybelline eyeliner, Maybelline lipstick, and Maybelline clear smooth all in one. The advertisement text for these cosmetics were taken from the Maybelline website: https://www.maybelline.co.id/.

### Data collection

Data were gathered using an observational methodology ( Tables 5- 7). The researcher examined the advertisements by searching for them on Google in order to download the complete text of the advertisements. The researcher then downloaded cosmetics advertising text that was in English. The procedures for gathering the data in this study make use of numerous strategies, including:

1. Searching text for cosmetic adverts on websites or the internet.
2. Reading and selecting the website that offers English-language text for cosmetic advertisements.
3. Downloading carefully chosen website text for beauty adverts, https://www.maybelline.co.id/, https://www.loreal.co.id/. The research is primarily focused on examining Maybelline New York advertisements, https://www.loreal.co.id/ was also used for comparison and data collection.
4. Choosing the data from the advertisement that uses English.
5. Examining the details or terminology used in cosmetic marketing.
6. Eliminating data which contains the same information.
7. Listing and giving codes to all the clauses by using the tables.

The additional methods and full data are available under *Underlying data* [ Herman *et al.,* 2022a].

**Table 5.** The percentage of types of mood ( Fang, 2016).

No	Types of moods	Number	Percentage
1	Declarative		
2	Interrogative		
3	Imperative		
	**Total**		

**Table 6.** The percentage of types of speech ( Fang, 2016).

No	Types of speech function	Number	Percentage
1	Statement		
2	Question		
3	Offer		
4	Command		
	**Total**		

**Table 7.** The realization of speech function based on mood ( Fang, 2016).

No	Types of speech function	Types of mood		
		Declarative	Interrogative	Imperative
1	Statement			
2	Question			
3	Offer			
4	Command			
	**Total**			

### Data analysis

The researcher then conducted an analysis of the data. Most qualitative researchers use data to describe events, explain what they mean, and gain an understanding of them. Since various strategies require various methods of analysis, this study will concentrate on interpersonal meaning.

Miles, Huberman and Saldana (2014:31-32) stated that analysis in qualitative research consists of three steps that occur simultaneously, namely;

1. Data condensation,
2. Data presentation, and
3. Conclusion drawing and verification.

In this study, the data were analyzed through the following steps:

1. *Data condensation*
  The method of selecting, concentrating, streamlining, abstracting, and changing the unprocessed data that results from field notes is known as data condensation. The procedure for condensing data is as follows:
  a. Selecting information from the website's text-based cosmetic adverts.
  b. Concentrating on English-language cosmetic commercials.
  c. Converting the data to be simplified into a clause. The clause consists of mood and speech function which is used in cosmetic advertisement.
  d. Evaluating the collected data which consists of mood and speech function used in cosmetic advertising.
  e. Analyzing the data by selecting each clause for the type of mood and speech function. Data selection is based on mood elements (subject and finite) residue elements (predicator and complement) and so forth all of them were realized in declarative, interrogative and imperative as descriptive analysis. Then, the speech function is manifested in statements, questions, commands and offered as a result of interpersonal meaning interpretive analysis.
2. *Data display*
  The next stage is to display the data after it has been compressed. A data perspective is a structured, condensed collection of information that facilitates processes like verification and conclusion-making. To make the data easier to understand for readers, researchers present the data in the form of tabular analysis. First, the information was categorized according to the mood types imperative, interrogative, and declarative. Second, knowing the realization of mood in speech function as interpersonal meaning in cosmetic advertisement text. Below are formulas and examples of analysis in tabular form:
$$N=f\left(x\right)/n\;x\;100\%$$
  N: percentage of type f(x): total types
  Frequency of the sub category n: total types of all categories.

3. *Drawing and verifying conclusions*
  One of the crucial steps in this investigation is coming to and verifying conclusions. This is a method of learning the study's findings. Here, the researcher draws a conclusion after thoroughly defining various mood and speech functions and learning how the text of cosmetic commercials realizes interpersonal meaning.

## Results and discussion

Based on the data analysis, there were five cosmetic Maybelline New York advertisements chosen. To conduct this research, there were 25 clauses to identify. The following analysis had been conducted from the perspective of the interpersonal meaning by analyzing its use of mood, speech function and how the speech function is realized in cosmetic Maybelline New York advertisements ( Tables 8- 18). It is explained as follows:

1. *Types of mood realization*
  There were three types of mood realized in five cosmetic Maybelline New York advertisements. The moods were realized by declarative mood, interrogative mood and imperative mood.
  The following data and analysis were described:
  a. Declarative mood
**Advertisement 1 – Loreal ad quote**
    From the mood structure above, the clause can be identified by the position of the subject (Milla) which is before the finite (is).
  b. Interrogative
    Yes-no interrogative is the order Finite before Subject
**Advertisement 2 – Maybelline ad quote** ( https://www.maybelline.co.id/) [ Herman *et al.,* 2022a]
    From the mood structure above, it can be identified by the position of the subject (the next) which is after the finite (is).
  c. Imperative
    Imperative mood is the clause when the predicator at the beginning of the sentence, with or without the subject which is usually “you”.
**Advertisement 3 – Loreal ad quote** ( https://www.loreal.co.id/ [ Herman *et al.,* 2022a]
    From the mood structure above, it can be found from the clause that the predicator (visit) is at the beginning of the sentence. We also find that there is no subject after the predicator.
    According to the data, there were 37 clauses chosen which contain declarative mood, interrogative mood and imperative mood. The percentage of mood will be explained in the table below:
2. *Types of speech function realization*
  There were four types of speech function realized in five cosmetic Maybelline New York advertisements. The speech functions were realized by statement, question, offer and command. The following data and analysis were described:
  a. Statement
    A statement is something that is said aloud or written down that conveys information. A statement may be neutral or adverse.
**Advertisement 4 – Maybelline ad quote** ( https://www.maybelline.co.id/)
*How to use Maybelline gel pencils that lasts up to 16 hours of wear*
    From the sentence above, we can see that the sentence provides information about how to use the Maybelline gel pencil product for up to 16 hours wear.
  b. Question
    A question is a technique to demand information in the form of an interrogative statement. It can be a yes-or-no question or an information query (wh-question). A question is an interrogative statement that is used to obtain information or ask an inquiry.
**Advertisement 5 – Loreal ad quote** ( https://www.loreal.co.id/) [ Herman *et al.,* 2022a]
*What’s next from paris?*
    From the sentence above, it shows that the advertisement is trying to find out whether the consumers already know or not about the latest product from Paris. This sentence also uses a wh-question that functions to seek confirmation from the consumers regarding knowledge of the latest product from Paris.
  c. Offer
    Offer is defined as a way to provide someone with a good or service. Offering something also conveys a willingness to provide or perform it.
**Advertisement 6 – Maybelline ad quote** ( https://www.maybelline.co.id/) [ Herman *et al.,* 2022a]
*Jourdan is wearing new eye studio lasting drama waterproof gel pencil in cashmere white*
    From the sentence above, we can see that the sentence provides information about how to use the new studio eye drama waterproof gel pencil in cashmere white.
  d. Command
    By requiring the listener to provide something, you can receive knowledge, goods, or services. Demanding goods and service through an imperative statement, whether it takes the form of a positive or negative command, is another way to use a command. The subject is not included in the command sentence.
**Advertisement 7 – Maybelline ad quote** ( https://www.maybelline.co.id/) [ Herman *et al.,* 2022a]
*Add color to your cheeks with fit me! Blush*
    From the sentence above, we can see that the sentence gives orders to consumers by adding color to the cheeks with fit me! Blush.
    According to the data, there were 25 clauses chosen which contain a statement, question, offer and command. The percentage of mood is explained in the table below:
3. *Realization of speech function based on mood*
  There were four speech functions realized based on mood: Statement realized by declarative mood, question realized by declarative mood and interrogative mood, offer realized by declarative mood and imperative mood, command realized by imperative mood.
  The following data and analysis were described as follows:
  a. Statement realized by Declarative Mood.
**Advertisement 8 – Maybelline ad quote** ( https://www.maybelline.co.id/) [ Herman *et al.,* 2022a]
**Speech function: Statement**
    The speech function is a statement because it gives information. From the sentence above, we can see that the sentence provides information about their product (Maybelline). Maybelline informs about the superiority of its product which is the 1st gel pencil with translucent gel base. It will make it easier for users to use or apply this product.
  b. Question realized by Declarative Mood and Imperative Mood.
**Advertisement 9 *–* Loreal ad quote** ( https://www.loreal.co.id/) [ Herman *et al.,* 2022a]
**Speech function: Question**
    The speech function is question, because it demands information. From the sentence above, it shows that the advertisement is trying to find out whether the consumers already know or not about the latest product from Paris. This sentence also uses wh-question that function to seek confirmation from the consumers regarding knowledge of the latest product from paris.
  c. Offer realized by Declarative Mood and Imperative Mood
**Advertisement 10 – Maybelline ad quote** ( https://www.maybelline.co.id/)
**Speech function: Offer**
    The speech function is offer, because it gives goods-and-services. From the sentence above, Maybelline makes Jourdan the artist of the product. Maybelline provides the fact that Jourdan uses new eye studio lasting drama waterproof gel pencil in cashmere white. But, this actually becomes a means to offer the consumers, if they want to be like Jourdan, Maybelline.
**Speech function: Offer**
    The mood is imperative, because there is a predicator at the beginning and no subject. The speech function is offer, because it gives good feel and services. It gives a recommendation to the consumer that “at maybelline.com” the consumer will get the tips from Maybelline experts.
  d. Command realized by Imperative Mood
**Advertisement 11 – Maybelline ad quotes** ( https://www.maybelline.co.id/)
**Speech function: Command**
    The mood is imperative because there is a predicator at the beginning and no subject. The speech function is command, because it demands goods and services. The advertisement persuades the consumers to buy the product as soon as possible.
    From the data, there were 37 clauses chosen from clauses which contain a statement, question, offer and command. Statement is realized by declarative mood, question is realized by declarative mood and interrogative mood, offer is realized by declarative mood and imperative mood, command is realized by imperative mood. The percentage of mood is explained in the table below:

**Table 8.** Milla is wearing colour riche nail in “now you see me” (530) and colour riche lipcolour in drumbeat red (310). ( **https://www.loreal.co.id/** **)** [ Herman *et al.*, 2022a; Fang 2016].

Milla	Is	wearing	*colour riche nail in now you see me (530) and colour riche lipcolour in drumbeat red (310).*
Subject	Finite	Predicator	Complement
**Mood: Declarative**		Residue	

**Table 9.** What is the next from Paris? ( Fang, 2016).

what	is	the next	from paris?
wh-question	finite	subject	
**Mood: Interrogative**			Residue

**Table 10.** Visit our digital mag: loreal.com laselection ( Fang, 2016).

visit	our digital mag: loreal.com laselection
predicator	complement
**Mood: Imperative**	Residue

**Table 11.** The percentage of mood realized in cosmetic Maybelline New York advertisements ( Fang, 2016).

No.	Types of mood	Number	Percentage (%)
1	Declarative	18	48.64
2	Interrogative	2	5.40
3	Imperative	17	45.94
	Total	37	99.98

**Table 12.** The percentage of speech function in cosmetic Maybelline New York advertisements ( Firmansah, 2015).

No	Types of speech function	Number	Percentage (%)
1	Statement	18	48.64
2	Question	2	5.55
3	Offer	7	19.44
4	Command	9	25
Total		36	99.99

**Table 13.** Gel pencil with translucent gel base: how does it perform? ( Fang , 2016).

The how	Is	our 1st gel pencil with translucent gel base for easy glide
subject	Finite	complement
**Mood: Declarative**		Residue

**Table 14.** What is the next from Paris? ( Fang, 2016).

what	is	the next	from paris?
wh-question	finite	subject	complement
Mood: Interrogative			Residue

**Table 15.** Jourdan is wearing new eye studio lasting drama waterproof gel pencil in cashmere white ( Fang, 2016).

Jourdan	Is	wearing	new eye studio lasting drama waterproof gel pencil in cashmere white
subject	Finite	predicator	complement
Mood: Declarative		Residue	

**Table 16.** Do get expert tips at maybelline.com ( Fang, 2016).

do	get	expert tips	at maybelline.com
finite	predicator	complement	circumstantial: place
**Mood: Imperative**			Residue

**Table 17.** Finish look with color sensational powder matte lipstick in cruel ruby ( Fang, 2016).

finish look	with color sensational powder matte lipstick in cruel ruby
predicator	complement
Mood: Imperative	Residue

**Table 18.** The realization of speech function based on mood in cosmetic Maybelline New York advertisement ( Firmansah, 2015).

No	Types of speech function	Types of mood		
		Declarative	Interrogative	Imperative
1.	Statement	11		13
2.	Question		2	
3.	Offer	5		1
4.	Command	3		7
	Total	19	2	21

## Discussion

Based on the research and the analysis of 811 pieces of data, 18 clauses were found which were declarative, 2 clauses were interrogative, 17 clauses were imperative. From this we can see that almost all declarative sentences are needed because declarative words are statements. Just like a statement sentence that must have a subject and a finite. Therefore, there are more declarative sentences than other types of mood. To enable the researcher, there were 37 clauses to identify. Types of mood realization is declarative, interrogative and imperative. The types of speech function realization in cosmetic Maybelline New York advertisements are statement, question, offer and command. 18 clauses were found which were statement, 2 clauses were found which were question, 7 clauses were found which were offer and 9 clauses were found which were command. If you want to find the implicit referential meaning you can use the Halliday theory revised by Matthiessen. Therefore, the purpose of the conclusions described above are to determine the type of mood structure in the interpersonal meaning embodied in Maybelline New York cosmetic advertisements, and to find out how the interpersonal meaning in the Maybelline New York cosmetic advertisement is realized **.**

## Conclusion

Based on the analysis of interpersonal meaning in cosmetic Maybelline New York advertisements, there are some conclusions:

1. In interpersonal meaning, clauses are analyzed from the mood structure which consists of mood elements and residue elements. From the analysis of interpersonal meaning through the mood structure found in the Maybelline New York cosmetics advertisements, there are 28 subjects obtained and 28 finite obtained. For residue elements, 17 predicates are obtained, 28 complements are obtained, 2 mood adjuncts are obtained. And there are 18 clauses (50%) as declarative mood, 2 clauses (5.55%) as interrogative mood and 16 clauses (44.41%) as imperative mood.
2. There are four types of speech functions, namely statements, questions, offers, and orders. There are 18 clauses (50%) as statements, 2 clauses (5.55%) as questions, 7 clauses (19.44%) as offers, and 9 clauses (25%) as orders. By understanding the function of speech, the researcher concludes that the function of speech in an advertisement is one of the moods that attracts someone to buy the product. This is one of the reasons for the researcher to examine the function of language in an advertisement.

Cosmetic brand Maybelline New York has been a leader in the beauty industry for over a century, and its advertisements have an immense influence on consumer culture. In 2018, their ads began to incorporate more meaningful interpersonal messages in an effort to challenge societal norms and beauty standards. By featuring models of all races, sizes, and genders, the ads highlighted the importance of inclusion and self-love. This shift in messaging was further highlighted by the company’s collaborations with celebrities and influencers who promote positivity and acceptance. The realization of these interpersonal meanings in the 2018 Maybelline New York advertising campaign was significant in terms of its impact on consumer culture, as well as its long-term effect on the beauty industry. This shift in messaging away from conventional beauty standards and towards inclusivity and self-love could be indicative of a broader societal shift towards acceptance and understanding. However, there is still a need for further research in this area to better understand the limitations and future research scope of this new messaging. This could include analyzing the impact of Maybelline’s campaign on consumer behavior and attitudes, as well as exploring how other beauty companies are responding to this shift.

## Data Availability

Figshare: RAW DATA.docx. https://doi.org/10.6084/m9.figshare.21757856.v3 ( Herman *et al.*, 2022a). This project contains the following underlying data:
- Types of Maybelline Products.docx (Cosmetic Maybelline New York Advertisements, Maybelline Products) Data are available under the terms of the Creative Commons Attribution 4.0 International license (CC-BY 4.0).
