# Supplementary material for: The realization of interpersonal meanings in cosmetic Maybelline New York in 2018 advertisements
Source: F1000Res. 2024 May 31;12:968. Originally published 2023 Aug 10. [Version 3] doi: 10.12688/f1000research.129750.3 (PMC11237818; doi:10.12688/f1000research.129750.3)
Supplement: Peer review history

# Reviewer report: Reviewer response for version 3

**DOI:** 10.5256/f1000research.167200.r297662

**Published:** 2024-07-10

**Authors:** Samreen Lodhi

**Affiliations:** 1 Department of Business Administration, Jinnah University for Women, Karachi, Sindh, Pakistan

The research is methodologically sound because the study design is appropriate and significant from both managerial implications and educational standpoint.

 The major aspect is that enough information is included in the parts dedicated to analysis to allow for peer replication and validation of results.

 It is suggested that the researcher may look into the generalizability dimension of the research findings. However, transparency  are ensured by providing all source data required for repeating the outcomes and the information gathered for the study provides strong support for the conclusions derived from the findings.

 The work integrates pertinent recent research into its clear and precise presentation of the subject matter.

 For these reasons, the paper is deemed scientifically sound as written.

Is the work clearly and accurately presented and does it cite the current literature?

Yes

If applicable, is the statistical analysis and its interpretation appropriate?

Yes

Are all the source data underlying the results available to ensure full reproducibility?

Yes

Is the study design appropriate and is the work technically sound?

Yes

Are the conclusions drawn adequately supported by the results?

Yes

Are sufficient details of methods and analysis provided to allow replication by others?

Yes

Reviewer Expertise:

NA

I confirm that I have read this submission and believe that I have an appropriate level of expertise to confirm that it is of an acceptable scientific standard.

# Reviewer report: Reviewer response for version 3

**DOI:** 10.5256/f1000research.167200.r285382

**Published:** 2024-06-10

**Authors:** Danang Satria Nugraha

**Affiliations:** 1 Sanata Dharma University, Yogyakarta, Indonesia

The paper is scientifically sound in its current form and only minor, if any, improvements are suggested."

 This determination is grounded on the following factors:

 1) The work is presented with clarity and precision and adeptly incorporates current literature.

 2) The study design exhibits appropriateness and holds academic significance.

 3) The methods and analysis are sufficiently detailed to facilitate peer replication.

 4) The minor statistical analysis and its interpretation are found to be fitting.

 5) All source data underpinning the results are provided to ensure full reproducibility.

 6) The drawn conclusions are well substantiated by the results obtained.

Is the work clearly and accurately presented and does it cite the current literature?

Partly

If applicable, is the statistical analysis and its interpretation appropriate?

Partly

Are all the source data underlying the results available to ensure full reproducibility?

Yes

Is the study design appropriate and is the work technically sound?

Yes

Are the conclusions drawn adequately supported by the results?

Partly

Are sufficient details of methods and analysis provided to allow replication by others?

Partly

Reviewer Expertise:

Theoretical Linguistics

I confirm that I have read this submission and believe that I have an appropriate level of expertise to confirm that it is of an acceptable scientific standard.

# Reviewer report: Reviewer response for version 1

**DOI:** 10.5256/f1000research.142459.r207877

**Published:** 2024-04-19

**Authors:** Danang Satria Nugraha

**Affiliations:** 1 Sanata Dharma University, Yogyakarta, Indonesia

This article offers a well-organized and comprehensive description of the problem, meeting current research needs. The feasibility and uniqueness of the research suggest its value and warrant further review. However, to enhance the introduction section, clearly articulating the research's uniqueness and feasibility is crucial.

 To improve the article, the following points should be considered:

1. A detailed gap analysis is necessary.
2. The manuscript needs to articulate the importance of the research better.
3. The rationale must be strengthened by providing arguments about the locus and discussion.
4. The results should include an interpretation of the findings.
5. The conclusion should answer the research question and relate to previous research/findings.
6. Though the literature review and previous studies illustrate and explain the adequacy of the reasons for the study, the focus of the object being discussed needs to be strengthened.
7. To strengthen the study, the accuracy and scope of the literature review should be improved by adding more information where needed.
8. The last paragraph of the method section requires further elaboration.

Is the work clearly and accurately presented and does it cite the current literature?

Partly

If applicable, is the statistical analysis and its interpretation appropriate?

Partly

Are all the source data underlying the results available to ensure full reproducibility?

Yes

Is the study design appropriate and is the work technically sound?

Yes

Are the conclusions drawn adequately supported by the results?

Partly

Are sufficient details of methods and analysis provided to allow replication by others?

Partly

Reviewer Expertise:

Theoretical Linguistics

I confirm that I have read this submission and believe that I have an appropriate level of expertise to confirm that it is of an acceptable scientific standard, however I have significant reservations, as outlined above.

# Response

**Published:** 2024-05-28

**Authors:** Herman Herman

**Affiliations:** Universitas HKBP Nommensen Pematangsiantar, Indonesia

I am pleased to present the updated research report on "Interpersonal Meanings in Cosmetic Advertising." Following valuable feedback and recommendations, several enhancements have been made to the manuscript to address the comments and improve the overall quality of the research.

 Firstly, the gap analysis has been thoroughly revised to provide a more detailed examination of the existing literature and to better articulate the uniqueness and feasibility of the research. By integrating existing theoretical frameworks and emphasizing the importance of the relationship between the author and the audience in advertising communication, the study aims to explore the structure of mood and speech functions in advertising communication. Additionally, the importance of the research has been articulated more effectively by highlighting the significance of analyzing language, mood structures, and speech functions in cosmetic advertising.

 The manuscript now emphasizes the practical implications for marketers, advertisers, and consumer behavior researchers, as well as the potential impact on advertising practices in the cosmetics sector. Furthermore, the rationale has been strengthened by providing in-depth arguments about the locus and discussion, including ethical considerations about language and communication strategies in cosmetic advertising.

 Lastly, the results have been interpreted to provide a comprehensive analysis of the findings, particularly focusing on the prevalence of declarative mood and statement speech functions in the analyzed cosmetic advertisements. These improvements aim to enhance the clarity, significance, and depth of the research, and I believe that the updated manuscript now effectively addresses the comments and suggestions provided.
